# Plant Growth Biostimulants, Dietary Feed Supplements and Cosmetics Formulated with Supercritical CO_2_ Algal Extracts

**DOI:** 10.3390/molecules22010066

**Published:** 2017-01-03

**Authors:** Izabela Michalak, Katarzyna Chojnacka, Agnieszka Saeid

**Affiliations:** Department of Advanced Material Technologies, Faculty of Chemistry, Wrocław University of Science and Technology, Smoluchowskiego 25, 50-372 Wrocław, Poland; katarzyna.chojnacka@pwr.edu.pl (K.C.); agnieszka.saeid@gmail.com (A.S.)

**Keywords:** algae, supercritical fluid extraction, bio-products, agriculture, cosmetics

## Abstract

The review paper presents the use of algal extracts as safe and solvent-free components of plant growth biostimulants, dietary feed additives and cosmetics. Innovative technology that uses extracts obtained by supercritical CO_2_ extraction, as a method of isolation of biologically active compounds from algal biomass, is presented. An important part of the complete technology is the final formulation of the product. This enabled realization of the further step which was assessment of the utilitarian properties of the extract-based products. The extracts were analysed for the presence of biologically active molecules (e.g., plant hormones, polyphenols) which provide useful properties such as antioxidant, antiviral, anti-inflammatory and antibacterial. The bio-products were tested in germination tests and underwent field trials to search for plant growth biostimulatory properties. Tests on animals (laying hens experiments) were conducted to assess pro-health properties of new dietary feed supplement. Another application were cosmetic formulations (dermatological tests). The results of the application tests were very promising, however further studies are required for the registration of the products and successful implementation to the market.

## 1. Introduction

Natural bioactive compounds are an alternative to synthetic components. They can be derived from algal biomass. Algae contain many groups of active compounds including: (1) polysaccharides e.g., alginate, fucoidan, laminarin, agar, carrageenan, mannan, porphyrin, ulvan etc.; (2) pigments e.g., phycobilins (phycocyanin, phycoerythrin), carotenoids (carotene and xanthophyll—fucoxanthin, astaxanthin, zeaxanthin, lutein) and chlorophylls; (3) polyphenols e.g., phlorotannin, catechin, flavonoids etc.; (4) lipids including polyunsaturated fatty acids—PUFA (e.g., EPA—eicosapentaenoic acid (C20:5, *n*-3), DHA—docosahexaenoic acid (C22:6, *n*-3), GLA—gamma-linolenic acid (C18:3, *n*-6)); (5) plant growth-promoting substances e.g., plant hormones (cytokinins, auxins, gibberelins, abscisic acid, ethylene), betaines, polyamines, sterols; (6) peptides and proteins, as well as vitamins and minerals. These compounds are characterized by antioxidant, anticancer, antiviral, anticoagulant, antidiabetic, anti-allergic, anti-inflammatory, antihypertensive and antibacterial properties and are described in detail in many papers (e.g., [1,2,3,4,5,6,7,8,9]).

**Figure 1:**
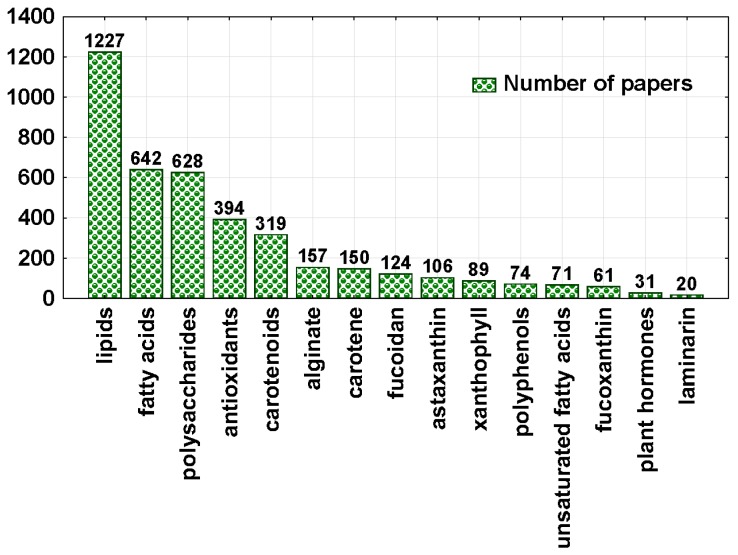
The most often extracted compounds from algae (according to the Web of Science; 27 October 2016).

We have checked in the Web of Science database (27 October 2016; http://apps.webofknowledge.com/), which algal biologically active compounds have received the most scientific attention. In the basic search, we used for example “extraction of polysaccharides from algae” in the topic (in the years 1926–2016). The obtained results are summarized in Figure 1.

Among the extracted compounds, lipids dominate (1227 scientific papers). Fatty acids (642) and unsaturated fatty acids (71) are also significant contributors. In the literature it is suggested that lipid extraction from algae (especially microalgae) has the highest potential for scale up and commercialization. The most promising area for the utilization of algal lipids is biodiesel production [10,11]. Second, the most studied group are polysaccharides (628) and among them alginate (157), fucoidan (124) and laminarin (20). Antioxidants, due to their unique properties constitute an interesting group of compounds (394). Special attention is paid to polyphenolic compounds (74) which exhibit a broad spectrum of beneficial biological properties such as antioxidant, anticancer, antimicrobial, anti-inflammatory and antidiabetic activities [12]. These interesting bioactive properties can be related to the presence of phlorotannins, which are complex polymeric phenolic compounds found only in brown seaweeds (Phaeophyta) [13,14]. Therefore, polyphenolic compounds are investigated for their potential use in food, cosmetic and pharmaceutical applications [12]. Algae are also a rich source of pigments because as photosynthetic organisms they can synthesise de novo chlorophyll and carotenoids. At present, there is a big demand for natural pigments e.g., in food products (dairy products, beverages, etc.), as feed additives, in cosmetics and in pharmaceuticals [15]. The main scientific interest is in carotenoids (319), which can be divided into xanthophylls (astaxanthin (106), xanthophyll (89), fucoxanthin (61)) and carotenes (150) [2,15]. Recently, algae have been also considered as a source of plant hormones (31) [16,17,18,19,20,21].

Generally, extraction techniques are used for isolation of these bioactive compounds and they include maceration, saponification, Soxhlet extraction with different organic solvents and also novel extraction techniques such as microwave-assisted (MAE), supercritical fluid (SFE), pressurized liquid (PLE) and ultrasound-assisted extraction (UAE) [14,22,23,24,25,26,27,28]. In the present paper we propose to use algal extracts obtained by supercritical fluid extraction as a safe and solvent-free components of plant growth biostimulants, dietary feed additives and cosmetics.

### Multiple Use of Algal Extracts—Concept of the Work

**Figure 2:**
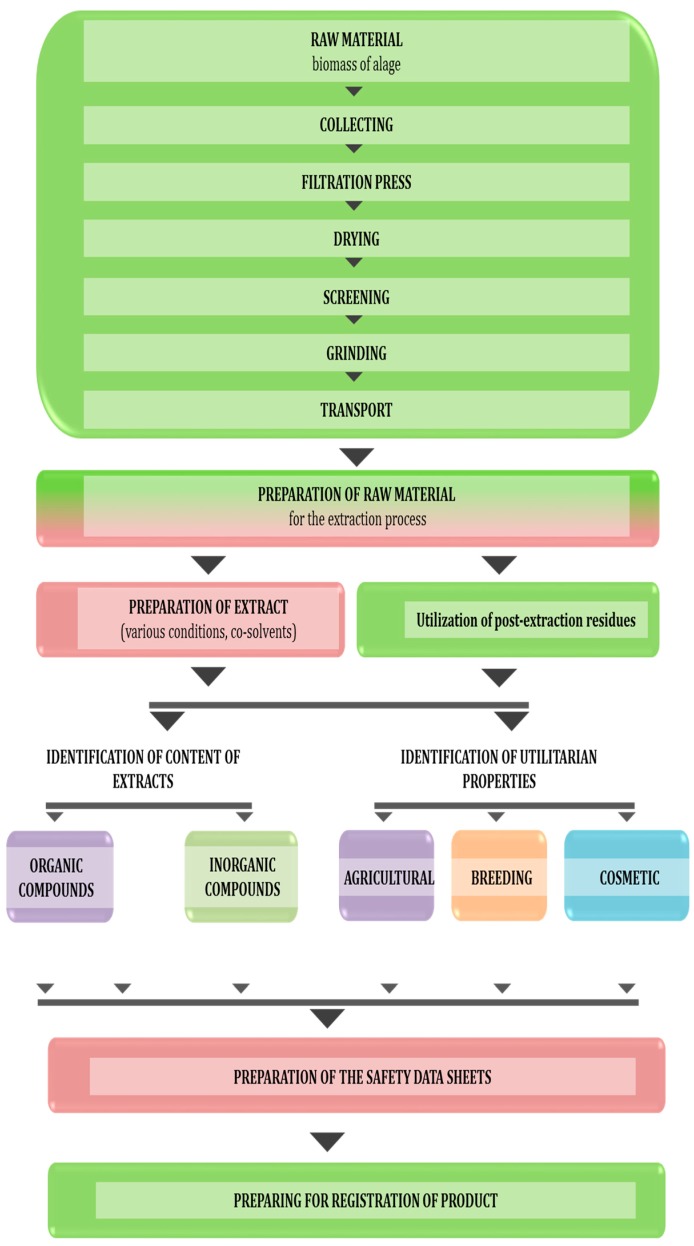
Using algae extracts as the components of fertilizers, dietary livestock supplements and cosmetics.

Algal extracts obtained by SFE, which is recognized as safe method by Food and Drug Administration (FDA) and European Food Safety Authority (EFSA), are known to be biodegradable, nontoxic, non-polluting and non-hazardous to humans and animals [26,29]. According to the Web of Science (2 June 2016), scientific interest overlaps with the increasing need for algal extracts. In the last 16 years, 309 scientific papers have been published whose topics featured such words and phrases as “application of algae extracts” and “food”, 97 with “cosmetics”, 42 with “fertilizers”, 10 with “biostimulant of plant growth” and six with “feed additives”. It is worth mentioning that until now, no successful commercial system for the production of supercritical algal extracts has been developed. There have only been some attempts to utilize SFE in the production of biofuels from microalgal biomass, because of high lipid contents of some algal species (over 60% *m*/*m*) [10]. The research strategy of the project carried out on supercritical extracts is presented in Figure 2.

In the present paper, special attention is paid to the application of algal extracts as plant growth biostimulants because this is a new solution to plant fertilization that has been investigated recently. Biostimulants are materials, other than fertilizers, that promote plant growth when applied in small quantities, boost mineral nutrient uptake and expand tolerance of plants to abiotic stresses, thus constituting an alternative to synthetic plant protection products [16,30]. In the recent years, the European Commission has completed the review of existing pesticides that were on the market before 1993. This programme concerned about 1000 substances and led to the withdrawal from the market of more than two thirds of these substances. All reviewed pesticides have undergone a detailed risk evaluation with respect to their effects on humans and on the environment (http://onlinelibrary.wiley.com/doi/10.1111/j.1529-8817.2011.00969.x/full; accessed on 3 June 2016). Moreover, on 1 January 2014, Europe acquired a new institution—the Integrated Pest Management (IPM). Professional users have to apply general principles of IPM. This are the promotion of low pesticide-input management including non-chemical methods. Algal extracts meet these requirements, which was also confirmed by the European Food Safety Authority [31].

The second area of our interest is the application of supercritical algal extracts as feed additives (especially added to drinking water of laying hens). According to the Web of Knowledge, there is no literature data concerning this issue. Algae have a high potential for the application in animal feeding due to the content of polysaccharides, proteins, PUFAs, polyphenols, pigments and minerals [32]. The Commission Regulation (EU) No 575/2011 of 16 June 2011 places algae on the list of feed materials placed.

Supercritical algal extracts can find also their application in the cosmetic industry. There is an increasing consumer awareness of products of natural origin, of their safety and quality. Algae are one of the most popular natural ingredients on the market [33]. They contain a variety of biological components with a broad range of physiological and biochemical characteristics that are rare or absent from other taxonomic groups, for example phlorotannins, pigments and sulphated polysaccharides [34]. At present, classical extraction methods are widely used for their isolation from algae [33,35].

## 2. Sources of Algae for the Extraction

The type of algal biomass used in the extraction process is a key element in the technology of algal extracts, which involves the isolation, identification of compounds extracted from biomass, preparation of formulation containing supercritical extract and the use of bio-products. Algal biomass for industrial purposes can be collected from the natural environment (marine and freshwater reservoirs), as well as can be acquired from special cultivations (e.g., microalgae in photobioreactors or in open raceway ponds) in order to obtain a homogeneous material. Cultivation and harvesting of microalgae is more complicated than in the case of macroalgae. A suitable harvesting method involves one or more steps and can be achieved by the use of several physical, chemical or biological processes in order to obtain the desired solid–liquid separation. Sedimentation, centrifugation, filtration, ultra-filtration are the most often used harvesting methods. In some cases some additional steps are applied, for example flocculation step or a combination of flocculation–flotation [36]. For example, Hong et al. proposed harvesting the microalgal biomass by chemical coagulation with 17% polyaluminum chloride, followed by dissolved air flotation [37]. In order to harvest microalgae from an open raceway pond, the biomass is allowed to settle by density for 2–3 h. Then, the biomass settled at the bottom is collected and fed to a bowl centrifuge [38].

Macroalgae (seaweeds) are easy to obtain because they are abundant in the environment, especially due to eutrophication process. For commercial use, algal species collected from the natural environment must be precisely identified as there are single or mixed species communities [39]. Algae harvested from natural water reservoirs produce a variety of useful compounds probably due to metabolic alterations induced by environmental stress conditions [7] such as light (essential for autotrophic organisms), temperature, water current and pH, as well as concentrations of nutrients [40]. Since algae have the ability to accumulate heavy metals, special care should be taken [2,41]. Algae cultivated under laboratory conditions are known to have constant composition. For example, Messyasz et al. (2015) showed that freshwater green macroalga *Cladophora* collected from its natural habitat might be a better source of amino acids for cosmetic purposes than the same alga grown in the laboratory [41]. This is a very important aspect from the economic point of view because cultivating algal biomass in artificial systems is more expensive than harvesting of macroalgae from their natural habitat. Additionally this is also a method of utilization of waste algal biomass being a result of the eutrophication.

In Poland, there are two main sources that provide algae. Green macroalgae can be collected from the Baltic Sea beaches, where they are washed up in large amounts (and are a nuisance especially in tourist resorts [42]) and from freshwater reservoirs (e.g., in Wielkopolska province, Poland). The Wetlands Algae Biogas (WAB) Project (http://wabproject.pl) data show that 400 tonnes of algal biomass were collected from the beach (164 tonnes) and sea (231 tonnes) in Sopot (Poland) in July, August and September 2011 alone. The main species were *Polysiphonia*, *Ulva* and *Cladophora* [17]. *Cladophora* Kützing (Chlorophyta) is also one of the largest genera of freshwater macroalgae that have a worldwide distribution. It accounts for over 40% of filamentous green algae. *Cladophora* commonly occurs as attached or free-floating forms in lakes, rivers and in small water bodies in Poland. About 10 species of *Cladophora* occur in Polish inland waters and among them *Cladophora glomerata* (L.) Kützing was recorded the most frequently [43]. It was found that the content of biologically active compounds (peptides, proteins, carbohydrates, vitamins, amino and fatty acids) in thalli of *Cladophora* from inland waters was equally high as that from the marine ecosystems. For commercial applications, the potential abundance of *Cladophora* in freshwater is one of its important factors [44]. The second source of biomass are commercially available microalgae (e.g., *Spirulina* sp.) which are a reliable source of active compounds because they can be cultivated in photobioreactors or in open pond raceway cultures on a large scale [7,36,37,38,45].

## 3. Extraction Techniques

The extraction techniques differ in the yield of extraction and the composition of the extract, which defines the potential applications of the final product. Due to low costs and relatively high efficiency usually solvent extraction with water, ethanol or hexane is used to isolate active compounds [29,46,47,48]. Traditional extraction is still used because it is cheap, simple and easy to scale up [26], although care is needed because organic solvents are flammable, often toxic and dangerous to the environment [46,49]. Since they can cause thermo-degradation of the extracted compounds [47] they must be separated from the final extract, especially if the product is to be used in food applications [50]. The recent regulations intended to eliminate the use of organic solvents because of the impact on health, safety and the environment were underlined by Wieczorek et al. [18].

At present, novel, environmental-friendly techniques such as enzyme-assisted, microwave-assisted, pressurized liquid, supercritical fluid, and ultrasound-assisted extraction are being developed [26]. For example, enzyme-assisted extraction (in which enzymes hydrolyze algal cell wall and facilitate the transfer of active substances to the liquid phase [48]) is mainly used for the extraction of antioxidants, carotenoids (xanthophyll−fucoxanthin), lipids (including PUFA), polysaccharides from the biomass of algae [26]. Microwave-assisted extraction allows for extraction of PUFA (e.g., DHA), polysaccharides (e.g., fucoidan), pigments (carotenoids−fucoxanthin), phenols, phytosterols, phytol, iodine and bromine [26]. Pressurized liquid extraction can be applied for the isolation of algal antioxidants (e.g., phenols), carotenoids (e.g., fucoxanthin), fatty acids and phytol [26], as well as amino acid, mineral, monosaccharides such as glucose, fructose, mannitol [23] and sulphated polysaccharides (e.g., fucoidan) [24]. For the extraction of carotenoids and chlorophyll, lutein, PUFA (e.g., DHA), major, minor and trace elements—ultrasound-assisted extraction can be used. Supercritical fluid extraction is applied mainly for the isolation of pigments (chlorophyll and carotenoids−astaxanthin, canthaxaothin, β-carotene, fucoxanthin), lipids (*n*-3: EPA and *n*-6 fatty acids: GLA) and vitamin E [2,14,22,26] and phlorotannins [14].

Among these techniques, special attention should be paid to supercritical fluid extraction by means of which not only solvent-free extracts but also compound fractionation can be achieved. Carbon dioxide, a non-toxic, inflammable, inexpensive and easily separable solvent has been more frequently used as a solvent in this extraction [51,52]. In a supercritical state (heated and pressurized), CO_2_ extracts biologically active compounds which due to a further reduction in pressure separate from CO_2_ [49,51]. CO_2_ has an additional ability to solve various substances by changing pressure and temperature during extraction that may be easily controlled [51]. The extraction conditions are mild: the critical temperature (31.1 °C) and pressure (72 bar) of CO_2_ are relatively low [52], which protects compounds that are sensitive to heat (the risk of thermal degradation is low) and aggressive chemicals [47,53]. The industry makes frequent use of an extraction pressure in SFE of ~35 MPa (350 bar). Working under pressures of >35 MPa may be economically advantageous, because as the pressure increases, the duration of the extraction process decreases and the amount of CO_2_ may also decrease significantly [54]. Response Surface Methodology (RSM) optimizes the production of the extract by SFE. The functional relationship between extraction responses (e.g., extract yield, content of the extracted compound, activity of extracts, for example antioxidant) and independent variables (extraction pressure, extraction temperature, ethanol content in supercritical CO_2_ extractions) must be determined [51,55].

SFE with CO_2_ is an appropriate method for the extraction of hydrophobic compounds (e.g., lipids). It is a non-oxidant medium that allows for the extraction of thermally labile or easily oxidized compounds, e.g., fatty acids [47,52]. Carbon dioxide cannot solve polar compounds, for example phenols, proteins, polysaccharides and minerals because of their insolubility in this fluid [52]. Lack of the extraction of elements from biomass (especially toxic elements) is beneficial for the cosmetic industry [17]. The addition of organic solvents (co-solvents) to supercritical CO_2_ at low concentrations (1%–10%), e.g., methanol, ethanol, acetone, acetonitrile, diethyl ether, dichloromethane or the addition of water increases efficacy of polar compound extraction [51,52,56]. Their function is to develop the solvating power of the fluid towards the target compound [27]. Numerous studies showed that ethanol used as a co-solvent is not very effective for the extraction of carotenoids, fucoxanthin and phlorotannin from seaweeds due to their different polarities. Saravana et al. proposed to use for their extraction sunflower, soybean and canola oil to support supercritical carbon dioxide extraction. The best results were obtained for sunflower oil as a co-solvent which showed higher fatty acid content, antioxidant activity and oil stability than the standard supercritical carbon dioxide extraction was used [14]. Table 1 presents examples of SFE extraction of biologically active compounds from algal biomass.

Reyes et al. proposed a new approach in that they found that the SFE with CO_2_ of astaxanthin from microalga *Haematococcus pluvialis* Flotow required long extractions at high pressures because this is a large molecule with low solubility in supercritical CO_2_. Therefore, in order to increase astaxanthin solubility in this fluid, ethanol was used as a co-solvent. Results showed that the ethanol content in CO_2_ (0%–13%, *w*/*w*) had a more significant effect on the yield than pressure (20–35 MPa) and temperature (40–70 °C). That is why they used CO_2_-expanded ethanol (ethanol content 50%–70%, *w*/*w*) at a fixed pressure (7 MPa) and temperature (30–60 °C). It turned out that temperature and ethanol content had a significant influence on astaxanthin yield [55]. Fujii (2012) examined the extraction of astaxanthin from microalgae using the conventional method (biomass soaked in ethanol and then centrifuged), SFE with CO_2_ and with the addition of ethanol. Ethanol as a co-solvent in SFE-CO_2_ improved the extraction yield of astaxanthin especially for the ratio biomass/solvent of 1 g/20 mL when compared with SFE-CO_2_ and the conventional method [57]. As it was shown in this study, an addition of ethanol to CO_2_ increased the lipid extraction from *Spirulina maxima* (Setchell & N.L. Gardner) Geitler from 32% to 40%, although the yields obtained are lower than those by ethanol (73%) and acetone (60%). The yield in SFE can be improved by the increase in the solvent/alga ratio. Opposite results were obtained by Li et al. who examined different extraction methods of fatty acids from marine microalga *Tetraselmis s*p. These methods included classical Bligh & Dyer lipid extraction (chloroform−methanol; 1:2), chemical extractions using different solvents (dichloromethane−methanol; 2:1, propan-2-ol−hexane; 1:1.25), direct saponification (96% ethanol + 3 nM KOH) and supercritical CO_2_ extraction. These extraction techniques produced significantly different yields and fatty acid compositions with SFE being the most effective for the extraction of microalgal lipids, especially long-chain unsaturated fatty acids [47]. Messyasz et al. also proved the superiority of supercritical fluid extraction of fatty acids from the biomass of *Cladophora glomerata* over the classical extraction method (Soxhlet). The highest fatty acid content in the dry matter of the extract was found for SFE (63%), then for extraction with acetone (35%) and finally with ethylene alcohol (19%) [41].

The main disadvantages of this process are high power consumption, high investment cost and labour-intensive step of sample processing [18,26]. Nevertheless, SFE can be successfully used as an alternative to traditional extraction methods, in particular when considering sustainability [53].

### Supercritical Fluid Extraction of Baltic and Freshwater Seaweeds and Microalgae

We elaborated a new technology for the preparation of algal biomass for supercritical fluid extraction. The secondary metabolites were isolated from the biomass of marine (*Polysiphonia*, *Ulva* and *Cladophora*), freshwater (*Cladophora*) and commercially available microalgae (*Spirulina* sp.) [66,67,68]. In the technology of algal extracts, each step of upstream processing (biomass cultivation/harvesting/drying, cell disruption), the process of extraction and downstream processing is equally important [69,70].

Downstream processing concerning the recovery and purification of compounds isolated from algae was discussed in the work of Wilk and Chojnacka. This step is indispensable in the manufacture of final, formulated products prior to putting them on the market. The technology consisted of the biomass collection, rinsing with tap water to remove salt and sand, drying, shredding and removal of the contaminations such as stones, sand, shells and pieces of wood [70]. Then the biomass was subjected to the final drying process, in the rotary drum at a temperature of below 55 °C, to reach a moisture of about 15%. The remaining fraction underwent a process of final purification from sand [17].

In order to increase the extraction yield of active compounds, very often different pre-treatment procedures of the biomass before extraction are applied. These methods involve cell disruption by mechanical, thermal, physical, chemical and enzymatic means [26]. Extraction requires deterioration of the structure of cell wall because it consists of polysaccharides, cellulose, hemicellulose and lignin [71]. Michalak et al. described supercritical fluid extraction from different forms of Baltic seaweeds. The best results (extraction yield) were obtained for the fine-grained algal grist produced after sieving the milled biomass (with particle size 0.25–20 mm) with a 0.5 mm sieve when compared with the pellets received using a granulator [17]. Mendes et al. also found that the degree of crushing of microalga *Chlorella vulgaris* Beyerinck (Beijerinck) influenced positively the extraction yield of carotenoids (astaxanthin, canthaxanthin). The best results were obtained for well crushed cells, then for slightly crushed cells and finally for whole cells. This increase can be put down to due to a higher accessibility of the supercritical fluid to the extracted compounds [56].

The supercritical fluid extraction of algae was performed in the New Chemical Syntheses Institute in Puławy, Poland. Supercritical Extraction Department has unique equipment for high pressure testing of extraction of natural raw material with the use of CO_2_. For the extraction of active compounds from algal biomass, 1/2—pilot plant equipped with two extractors with a capacity of 40 L each was used. This plant works under a pressure of up to 100 MPa (1000 bar) and at a temperature of up to 100 °C (for further details see Rój [51]). The best experimental conditions for the supercritical fluid extraction with CO_2_ of Baltic, freshwater seaweeds and microalgae (*Spirulina* sp.) were determined. For example, for the Baltic seaweeds, the experimental conditions were as follows: pressure 500 bar, temperature 40 °C, CO_2_ amount per kg of load mass—79 kg CO_2_. In these conditions the extraction capacity was 1.8% [17]. Biologically active compounds were extracted also from freshwater *Cladophora glomerata* at 700 bars and at 45 °C [41]. In the case of *Spirulina* sp. the supercritical extraction was performed at a pressure of 700 bar and at a temperature of 40 °C. Raw material extraction time was 8 h and extraction yield was 5.6% [72]. In our work it was confirmed that several factors influenced the supercritical extraction yield, in particular: temperature, pressure and the presence of a polar co-solvent (methanol, ethanol, methanol–water mixtures) [66,73]. Therefore, the optimization of this process was found to be crucial [51]. Wilk et al. reported that the SFE with a co-solvent (ethanol) was more effective than without and resulted in the increased extraction yield from brown alga *Fucus* sp. [66].

## 4. Analytical Methods Used for the Identification of Compounds Extracted from Algae

In order to implement algal extracts as a component of the commercial products on the industrial scale, detailed chemical and biological analysis is required [74]. However, the complexity of isolation, purification and identification of these substances still seems to limit the use of this source of natural chemicals, hence an elaboration of analytical procedures is essential to the standardization of algal bio-products [39].

Several analytical methods such as Thin Layer Chromatography (TLC), High-Performance Liquid Chromatography (HPLC), UV-VIS can be used for the determination of biologically active compounds present in the extracts [18,19,51]. Rój demonstrated that most useful was TLC, which was often treated as a screening method for HPLC [51]. Michalak et al. examined supercritical algal extracts in terms of the content of plant hormones such as auxins and cytokinins by HPLC, polyphenols by spectrophotometry, lipids including fatty acids by gas chromatography (GC), macro- and microelements by Inductively Coupled Plasma-Optical Emission Spectroscopy (ICP-OES) [17]. Górka et al. developed and optimized a new High-Performance Liquid Chromatography with Photodiode Array Detection (HPLC-PDA) method. After some modifications, it was possible to separate and determine simultaneously nine plant hormones in algal extracts that derived from different classes of compounds. The following phytohormones were detected: indoleacetic acid (IAA), indolebutyric acid (IBA), phenylacetic acid (PAA), naphthaleneacetic acid (NAA), *trans*-zeatine (TZ), kinetin (KA), isopentenyladenine (IA), benzylaminopurine (6-BA), abscisic acid (ABA) [75].

## 5. Algal Extracts as Plant Growth Biostimulants

Biomass of algae contain many molecules which are active at various stages of plant growth. In agriculture, algae can be used in different forms, of which extracts appear to be the most effective [16,76]. Examples of commercially available algal based products for agriculture are presented in the work of Michalak and Chojnacka [77]. For the first time, algal extracts were used in modern agriculture about 60 years ago. Currently, commercially available algal extracts are liquids from green to light brown and slightly acidic. The nature and concentration of the bioactive compounds in algal extracts depend on their structure and content in algal cells, but first of all on the method of their production. Proper selection of the conditions under which extraction is performed plays a key role [19]. The information about compounds that promote plant growth, especially about methods of their chemical analysis and application of algal extracts into processes of maintaining plant crop, are essential to the elaboration of innovative products for agriculture [30,53].

**Figure 3:**
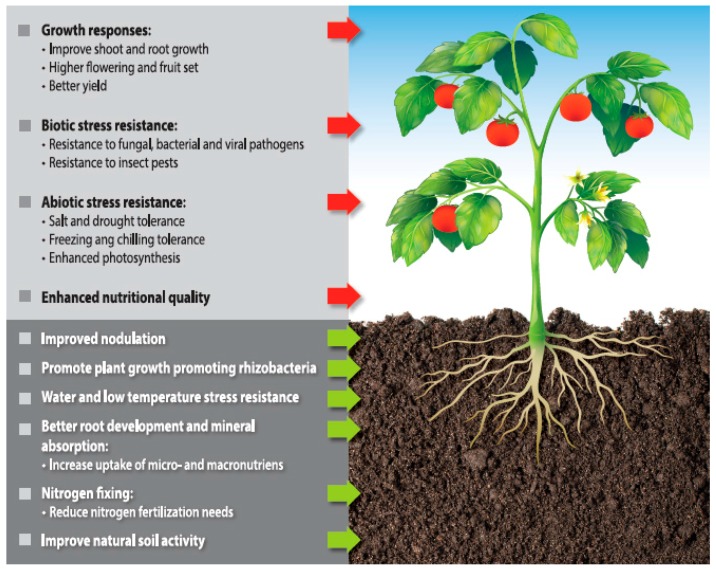
Possible effect of algal extracts.

Agriculture makes extensive use of extracts from *Ascophyllum nodosum* (Linnaeus) Le Jolis, *Fucus* spp., *Laminaria* spp., *Sargassum* spp. and *Turbinaria* spp. [16]. The main chemical components of seaweed that affect plant growth are phytohormones (cytokinins, auxins and auxin-like compounds, indole acetic acid, abscisic acid), betaines, sterols, polysaccharides, minerals and trace elements, amino acids, polyphenols (e.g., phlorotannins) [16,18,19,29,74,75]. Plant hormones seem to be the most essential for enhancing the growth and development of crop plants [19,20,75]. Algal extracts stimulate not only plant growth, but also plant mass, the content of chlorophyll, carotenoids and improve root development [16,77]. The possible effect of action of algae extract is presented in the Figure 3.

Algal extracts are also known to protect plants against pathogens and insects (resistance of plants to diseases) and against abiotic stress (high salinity, drought, frost etc.) [16], which is why they can be an alternative to chemical pesticides. As an example, marine macroalgae contain polysaccharides that trigger signaling cascades, which activate plant defense response and resistance to infection by pathogens. This property is directly related to a degree of sulfatation. Natural elicitors of plant defense are laminarins (from brown algae), ulvans (from green), carrageenans (from red) and fucans (from brown) [21]. Phlorotannins, another type of compound, protect cells from oxidative stress and radiation-induced injury and can be a natural alternative to synthetic chemicals [78]. Algal polysaccharides (mainly alginates, fucoidans, carrageenans and laminarins) are also characterized by their possible chelating properties. These molecules have a high capacity to bind cations of trace elements, following the presence of a large number of chemical groups, mainly carboxyl residues. This function allows algal extracts to be used as a foliar fertilizers with micronutrients. Additionally, these hydrocolloids possess gelling and water-binding capacity [79].

There are many examples of the application of algal extracts as plant growth biostimulants, e.g., [16,17,68,80,81], etc. However, most of them were produced by conventional extraction techniques. Few studies have been conducted on supercritical algal extracts. Before their application as a foliar or soil spray, liquid formulations must be obtained [67,68,76]. Such a formulation should contain algal extract (as a source of the active ingredient), wetter, co-dispersant, dispersing agent, antifreeze, preservative and solvent [72,82]. Michalak et al. have shown that supercritical extracts obtained from Baltic seaweeds contained inorganic (macro- and microelements), as well as organic compounds such as plant hormones (auxins, cytokinins and polyphenols) that can influence plant metabolism. The supercritical extraction method yields small amounts of toxic elements from raw algal biomass [17].

The utilitarian properties of the algal supercritical extracts were examined in the germination tests on cress (*Lepidum savitum* L.) [67,68] as well as in field trials on winter wheat (variety *Tacitus*) [72,83]. The results of germination tests showed that the formulations containing algal extract from Baltic seaweeds and *Spirulina* sp., applied in doses of 0.009–0.045 mL per Petri dish, were superior to control samples (11%–80% fresh sprout mass increase) and similarly effective to commercial preparation (Kelpak SL [68]). It was also found that seed treatment of wheat with supercritical formulation containing Baltic seaweeds improved root development. The best sprout growths were achieved for seeds coated with 8 μL of *Spirulina* sp. supercritical formulation. Wheat seeds (1 g) were coated with three doses (8, 14 and 20 μL) of a formulation containing supercritical CO_2_ extract from *Spirulina* sp. or Baltic seaweeds. The treated seeds were cultivated on cotton. The fresh sprout mass, sprout heights and root lengths were determined vs. control. The coating resulted in increasing the mass yield and the sprout height by approximately 13% and 7%, respectively [67].

The stimulatory affect in the initial growth phase of plants was also checked for algal homogenates which were used for seed coating. Baltic dried seaweed (*Enteromorpha* sp. and *Cladophora* sp.) was homogenized and two emulsion concentrates were prepared, one of which was enriched with minerals (Mn, Mo, Zn, Mg, Cu and Fe). Both formulations were tested in assays of spring wheat seed germination at doses of 10, 25 and 50 μL per Petri dish. The utility of seed coating was verified by measurements of fresh weight of plants and root length of sprouts, as compared to untreated crops. Each of the applied doses of the formulation proved to affect both sprout and root development. Future research will also check the effect of algal homogenate against most typical conditions of abiotic and biotic stress [84]. Świniarska et al. examined the toxicity of the supercritical *Spirulina* sp. extract on plants (rapeseed and mustard) and on *Eisenia fetida* (Savigny, 1826) earthworms. The extract and emulsifier were added to soil samples at different ratios. In both cases the best plant growth stimulation was achieved at a mass ratio of 1:1 and 1:100 (extract−emulsifier). For earthworms, after a 14-day test, no difference in body mass the earthworms or their mortality was observed [85].

Field trials on winter wheat (variety *Tacitus*) (2013) showed that the supercritical extract from *Spirulina* sp. (applied in two doses—1.20 L/ha and 1.8 L/ha) slightly increased the crop yield per hectare in comparison with the control group—untreated (4.0%) and the reference product, Asahi SL applied at a dose of 0.60 L/ha (2.0%). The choice of doses was dependent on the content of polyphenols in the applied products [72]. Similar effect was observed in the case of the application of supercritical extract obtained from brown seaweed—*Ascophyllum nodosum* (dose 1.0 L/ha), Baltic green macroalgae (dose 1.0 L/ha) and microalga *Spirulina* sp. (doses 1.0, 1.5 and 1.8 L/ha) in the next growing season (2015). Among the tested supercritical extracts, the best results in terms of grain yield, number of grains in ear were obtained for *Spirulina* (1.5 L/ha) [83].

## 6. Algal Extracts as Components of Cosmetics

Algae are a valuable raw material for cosmetics production due to the presence of variety of phytochemicals such as polysaccharides (e.g., alginates, carrageenans, ulvans, fucoidans, laminarans, agar), proteins and their derivatives (e.g., amino acids, lectins, cyclic peptides), lipids (e.g., fatty acids, galactoglycerolipids, phytosterols), pigments (e.g., chlorophylls, carotenoids, phycobiliproteins) and phenolic compounds (e.g., phlorotannins, bromophenols, terpenoids) [8,33,34]. Polysaccharides are mainly used as thickeners, emulsion stabilizers, protective colloids, gelling, moisturizing and chelating agents. They possess antioxidative, anticellulite, antiviral, anti-inflammatory, antiaging properties. Proteins and their derivatives have antioxidant, chelating, antibacterial, antiviral, antifungal and anti-inflammatory properties. They can also act as moisturizers and natural sun screeners. Algal lipids, phenolic compounds and pigments are antioxidant, antiaging, anti-wrinkle, anti-allergic, anti-inflammatory, antimicrobial and regenerating components. Phenolic compounds act also as natural UV screens [8,33,34,86,87,88].

In cosmetics, algae can be used in micronized form or as extracts. They can be a component of antiaging or moisturizing face creams, serum, antiacne cosmetics, peelings, body scrubs, anticellulite body lotions and masks or slimming body creams [33,88]. It should be underlined that commercialization of algal-based cosmetics is a complex issue and requires testing of the products in clinical studies. Products based on algae should be prepared in accordance with the set of rules and standards. Algae products cannot easily be out on the market since there are no strict guidelines on the assessment of their efficacy and safety. A multi-technique and multi-stage approach is required in order to obtain the high value and safety of algae-based products for the cosmetic industry [33].

Nowadays, extracts of algal biomass produced with the classical methods of extraction with a suitably selected solvent (ethyl alcohol, propylene glycol, vegetable oils) are the primary sources of bioactive components for the cosmetics industry. However, as it was shown in the work of Messyasz et al., SFE with carbon dioxide which is considered as a “green solvent” can be more efficient in the extraction of biologically active compounds than conventional techniques. It was shown that the fatty acid content in the dry matter of the extract obtained from freshwater *Cladophora glomerata* varied depending on the extraction method used: ethylene alcohol (19%), acetone (35%) or supercritical fluid extraction (63%) [41]. Schroeder et al. discussed process optimization for the production of supercritical algal extracts in three areas: biomass harvesting from the natural environment, extraction with carbon dioxide under supercritical conditions and formal requirements, enabling the use of an extract for commercial purposes as a component of cosmetic preparations. Characteristics of algal extracts need yet to be examined [25].

## 7. Algal Extracts as Components of Feed Additives

Korczyński et al. indicated that currently there is a need to search for new feed additives that will contribute to high quality products and farm animal welfare [32]. Among the materials of natural origin, seaweeds may play the role of functional fodder additives. As it was mentioned, the regulation of European Union (No 575/2011, 16 June 2011) places algae in the list of feed materials. Algae can be fed to animals because they contain polysaccharides such as fucoidan and laminarin, which are known to possess antibacterial properties and have a positive effect on intestinal microflora, as well as essential amino acids, proteins (e.g., lectins), essential unsaturated fatty acids, especially from group *n*-3, micro- and macroelements, polyphenols and pigments that exhibit strong antioxidative activity (treatment and prevention of numerous diseases) and can additionally to improve the coloration of animal origin products [32,89].

In animal feeding algae can be used in different forms, raw or processed (including fresh, chilled or frozen algae); dried algae; as algae meal (product of algae oil manufacture, obtained by extraction of algae); algal oil (product of oil manufacture from algae obtained by extraction); algae extract (watery or alcoholic extract of algae that principally contains carbohydrates) (European Union, No 575/2011, 16 June 2011). Among these, algae extracts (especially supercritical algal extracts) are the least examined in animal feeding. Most works concern the application of extracts (containing β-glucan, laminarin, fucoidan) from brown macroalgae (*Laminaria digitata* (Hudson) J.V. Lamouroux, *Laminaria hyperborean* (Gunnerus) Foslie, *Ascophyllum nodosum*) in pig nutrition [2]. The application of supercritical algal extracts to drinking water of laying hens is a new approach proposed by Korczyński et al. (2015). Algal extracts applied in feeding experiments of animals could contribute to the biofortification of the products of animal origin in biologically active compounds [32].

## 8. Possible Commercialization of Supercritical Algal Extracts

Mainly microalgae are well-suited for commercialization [45,90]. Their biomass is considered to be a rich source of energy and liquid fuels [9,10,36,37,73]. There are some global companies involved in the commercialization of high-value compounds from algae, for example Solarvest BioEnergy and Canadian Pacific Algae in Canada, Algomed and BlueBioTech in Germany, Algatechnologies and UniVerve Biofuel in Israel, AlgaeLink N.V, AlgaeBiotech and LGem in The Netherlands, Cellena, Cyanotech, Algaeon and Alltech Algae in USA (see more in [90]). Various valuable compounds can be extracted from microalgal biomass, for example phycocyanin (*Galdieria suphuraria* (Galdieri) Merola, *Arthrospira platensis* Gomont (=*Spirulina platensis*)), β-carotene (*Coelastrella striolata* var. *multistriata*, *Coccomyxa acidophila*, *Dunaliella salina*, *Scenedesmus almeriensis*), astaxanthin (*Coelastrella striolata* var. *multistriata* (Trenkwalder) Kalina & Puncochárová, *Haematococcus pluvialis*), fatty acids (*Diacronema vlkianum* Prauser, *Crypthecodinium conch* (Seligo) Javornicky, *Isochrysis galbana* Parke, *Microchloropsis gaditana* (L.M. Lubián) M,W. Fawley, I. Jameson & K.P. Fawley (=*Nannochloropsis gaditana*), *Odontella aurita* (Lyngbye) C. Agardh, *Phaeodactylum tricornutum* Bohlin, *Schizochytrium* sp., *Ulkenia* spp.). They are used as bulk commodities and specialty chemicals in different industrial sectors (e.g., pharmaceuticals, food supplements, functional foods, feed additives, cosmetics, nutraceuticals, fuel production (biofuels)) [90]. *Dunaliella salina* was the first alga which was commercialized as a source of β-carotene [45].

A supercritical fluid extraction of compounds from algal biomass has not been put on the market yet. Further research concerning algae harvesting, extraction process, optimization of residual biomass utilization is required for the successful implementation of this technology on a technical scale. Molina Grima et al. assessed that in microalgal biotechnological processes the downstream processing can account for 50%–80% of total production costs, depending on the biochemical characteristics of the compound and the purity ratio that needs to be achieved [91].

In our previous work it was assessed that the cost of extracting one tonne of biomass is about €1500 [17]. This price includes the cost of CO_2_, steam, electrical energy, depreciation, personnel, cleaning, repairs and others. For the equipment used in the present study, depreciation constituted the main cost. However, the produced algal extract is in concentrated form of biologically active compounds and only small amounts are added to the final products. In the future, new raw materials, with increased content of hydrophobic biologically active compounds will be selected for the supercritical fluid extraction.

## 9. Conclusions

Since chemical synthesis of biologically active compounds is difficult, it is important to search for natural bioactive compounds and their isolation techniques. Designing natural-derived products that yield the desired outcome still remains a challenge. Active compounds are synthesized by cells in low quantities and are present as conjugates or mixtures in extracts. Production, detection, separation, purification and characterization of components of algal extracts that could find application is essential if the products are to be introduced on the market. Purification and standardization procedures should be elaborated. In many countries, the system of algal biomass collection from water-bodies has not been implemented yet. It is essential for successful large-scale use of algae biomass as a raw material for industrial and agricultural development of products. The present review reports also data on the investigation of their utilitarian properties. There is a chance to implement new bio-products from algae by supercritical fluid extraction to the market, because they constitute a concentrate of biologically active compounds without the presence of organic solvents. The results of applicative studies are promising. The tests on laying hens confirmed the beneficial effect on laying efficiency, the properties of eggs (biofortification with polyunsaturated fatty acids) and health condition of hens. The field studies showed good biostimulatory effect, related with biofungicidal properties. Resulting, higher yield of crops by 10%–15% was observed. Another useful application were cosmetic formulations. Anti-wrinkle and hydrating properties were confirmed under in vitro and in vivo conditions.

## Figures and Tables

**Table 1:** Examples of SFE extraction of biologically active compounds from algal biomass.

Extraction	Algae	Temperature (°C)	Pressure (bar)	Extraction Time (min)	Extracted Compound	Reference
PIGMENTS						
SFE with CO_2_	*Undaria pinnatifida* (MA)	25–60 (40 *)	200–400 *	180	fucoxanthin	[58]
SFE with CO_2_ and with EtOH	*Saccharina japonica*, *Sargassum horneri* (MA)	45	250	120	fucoxanthin	[22]
SFE with CO_2_ and with sunflower oil/soybean oil/canola oil/ethanol/water	*Saccharina japonica* (MA)	45–55	200–300	120	fucoxanthin	[14]
SFE with CO_2_	*Dunaliella salina* (MI)	40, 60	200	n.a.	β-carotene	[56]
SFE with CO_2_	*Chlorella vulgaris* (MI)	40 *, 55	150, 200, 275, 350 *	n.a.	total carotenoids, astaxanthin, canthaxanthin	[59]
SFE with CO_2_	*Chlorella vulgaris* (MI)	40, 55	100–350 (275 and 350 *)	n.a.	carotenoids (astaxanthin, canthaxanthin)	[56]
SFE with CO_2_	*Dunaliella salina* (MI)	40, 50, 60 *	100, 200, 300 *, 400, 500	180	carotenoids, chlorophylls	[60]
SFE with CO_2_ SFE with CO_2_ and with EtOH	*Haematococcus pluvialis* (MI)	30 *, 45, 60	200	15 *, 30, 60	chlorophyll, astaxanthin	[57]
SFE with CO_2_ and with EtOH	*Undaria pinnatifida* (MA)	30, 40, 50 *, 60	80, 100, 150, 200 *, 250, 300	50	fucoxanthin	[61]
POLYPHENOLS						
SFE with CO_2_	*Polysiphonia, Ulva, Cladophora* (MA)	40	500	320 *, 360, 810	polyphenols	[17]
SFE with CO_2_ (modified with 12% EtOH)	*Sargassum muticum* (MA)	60	152	90	polyphenols	[28]
SFE with CO_2_ and with EtOH	*Undaria pinnatifida* (MA)	30, 40, 50, 60 *	80, 100, 150, 200, 250 *, 300	50	polyphenols	[61]
LIPIDS						
SFE with CO_2_	*Sargassum hemiphyllum* (MA)	40 *, 50 *	241–379 *	60	fatty acid profile of lipids (*n*-3: C18:3, C18:4, C20:5, C22:5, C22:6)	[62]
SFE with CO_2_	*Hypnea charoides* (MA)	40–50 *	241–379 *	120	fatty acid profile of lipids (*n*-3: C18:3, C18:4, C20:4, C20:5, C22:5, C22:6)	[63]
SFE with CO_2_	*Chaetomorpha linum* (MA)	50	260	420	oil	[64]
SFE with CO_2_	*Bangia atropurpurea*, *Porphyra angusta*, *P. dentate* (*Pyropia dentate*), *Helminthocladia australis, Liagora orientalis* (*Izziella orientalis*), *Liagora boergesenii, Scinaia monoliformis, Tricleocarpa cylindrica* (*Galaxaura cylindrical*), *Grateloupia filicina, Halymenia microcarpa (H. ceylanica)* (MA)	55	34.5	180	fatty acid (e.g., eicosapentaenoic acid, EPA, C20:5, *n*-3)	[9]
SFE with CO_2_	*Chlorella vulgaris* (MI)	40, 55	150, 200, 275, 350	n.a.	lipids	[59]
SFE with CO_2_	*Cladophora glomerata* (MA)	45	300, 500, 700	n.a.	polyunsaturated, saturated fatty acid	[41]
SFE with CO_2_	*Chlorella vulgaris* (MI)	40 *–70	200–280 *	540	lipids (saturated, mono- and polyunsaturated fatty acids)	[65]
SFE with CO_2_	*Tetraselmis* sp. (MI)	40	150	720	lipids (saturated, mono- and polyunsaturated fatty acids)	[47]
SFE with CO_2_ SFE with CO_2_ and with EtOH (10%)	*Arthrospira maxima* (*Spirulina maxima*) (MI)	(1) 50 (2) 50 *, 60	(1) 250 (2) 250 *	n.a.	fatty acids: γ-linolenic acid (GLA, C18:3, *n*-6)	[56]
PLANT GROWTH PROMOTING SUBSTANCES						
SFE with CO_2_	*Polysiphonia, Ulva, Cladophora* (MA)	40	500	320 *, 360, 810	auxins, cytokinins	[17]
MICRO- AND MACROELEMENTS						
SFE with CO_2_	*Polysiphonia, Ulva, Cladophora* (MA)	40	500	320 *, 360, 810	micro- and macroelements	[17]

n.a.: not available; EtOH: ethanol; MA: macroalgae; MI: microalgae; *: the most suitable conditions for SFE.
